# Preclinical Pharmacokinetics and Pharmacodynamic Target of SCY-078, a First-in-Class Orally Active Antifungal Glucan Synthesis Inhibitor, in Murine Models of Disseminated Candidiasis

**DOI:** 10.1128/AAC.02068-16

**Published:** 2017-03-24

**Authors:** Stephen A. Wring, Ryan Randolph, SeongHee Park, George Abruzzo, Qing Chen, Amy Flattery, Graig Garrett, Michael Peel, Russell Outcalt, Kendall Powell, Michelle Trucksis, David Angulo, Katyna Borroto-Esoda

**Affiliations:** a SCYNEXIS, Inc., Jersey City, New Jersey, USA; b Merck & Co., Inc., Rahway, New Jersey, USA

**Keywords:** SCY-078, candidemia, fungal disease, pharmacokinetics

## Abstract

SCY-078 (MK-3118) is a novel, semisynthetic derivative of enfumafungin and represents the first compound of the triterpene class of antifungals. SCY-078 exhibits potent inhibition of β-(1,3)-d-glucan synthesis, an essential cell wall component of many pathogenic fungi, including Candida spp. and Aspergillus spp. SCY-078 is currently in phase 2 clinical development for the treatment of invasive fungal diseases. *In vitro* disposition studies to assess solubility, intestinal permeability, and metabolic stability were predictive of good oral bioavailability. Preclinical pharmacokinetic studies were consistent with once-daily administration to humans. After intravenous delivery, plasma clearance in rodents and dogs was low, representing <15% and <25% of hepatic blood flow, respectively. The terminal elimination-phase half-life was 5.5 to 8.7 h in rodents, and it was ∼9.3 h in dogs. The volume of distribution at steady-state was high (4.7 to 5.3 liters/kg), a finding suggestive of extensive tissue distribution. Exposure of SCY-078 in kidney tissue, a target organ for invasive fungal disease such as candidiasis, exceeded plasma by 20- to 25-fold for the area under the concentration-time curve from 0 h to infinity (AUC_0–∞_) and *C*_max_. SCY-078 achieved efficacy endpoints following oral delivery across multiple murine models of disseminated candidiasis. The pharmacokinetic/pharmacodynamic indices *C*_max_/MIC and AUC/MIC correlated with outcome. Target therapeutic exposure, expressed as the plasma AUC_0–24_, was comparable across models, with an upper value of 11.2 μg·h/ml (15.4 μM·h); the corresponding mean value for free drug AUC/MIC was ∼0.75. Overall, these results demonstrate that SCY-078 has the oral and intravenous (i.v.) pharmacokinetic properties and potency in murine infection models of disseminated candidiasis to support further investigation as a novel i.v. and oral treatment for invasive fungal diseases.

## INTRODUCTION

Invasive fungal diseases represent a growing threat, and their incidence, mortality, and treatment recommendations have recently been reviewed in the 2016 *Clinical Practice Guideline for the Management of Candidiasis* (1). Here, the guideline authors summarize that over the past decade there has been a significant increase in the incidence of documented invasive infections caused by Candida species, to the extent that in many U.S. hospitals, candidemia represents the third or fourth most common hospital-acquired bloodstream infection. The increase in the incidence of invasive candidiasis has been driven by the rising number of critically ill patients who have widespread exposure to risks such as central venous catheters, broad-spectrum antibiotics, surgical procedures, and immunosuppressant medication. Although three classes of antifungals (echinocandins [ECHs], azoles, and polyenes) are available, a significant unmet need remains for patients with invasive fungal infections due to the increasing frequency of resistance to these therapies, in particular the emergence of multidrug-resistant strains and the lack of therapeutic options that allow for oral (p.o.) administration. Sadly, the overall mortality rate of invasive candidiasis remains high despite therapy.

**FIG 1:**
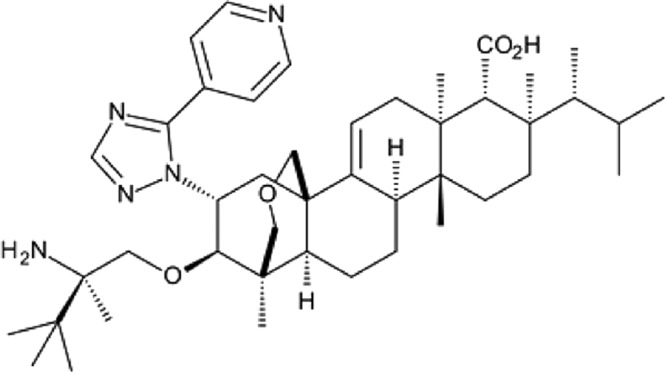
Chemical structure of SCY-078. Position 15 of the core phenanthropyran carboxylic acid ring system is modified with a pyridine triazole, and position 14 is modified with a 2-amino-2,3,3-trimethyl-butyl ether, (1*S*,4a*R*,6a*S*,7*R*,8*R*,10a*R*,10b*R*,12a*R*,14*R*,15*R*)-15-[[(2*R*)-2-amino-2,3,3-trimethylbutyl]oxy]-8-[(1*R*)-1,2-dimethylpropyl]-14-[5-(4-pyridinyl)-1*H*-1,2,4-triazol-1-yl]-1,6,6a,7,8,9,10,10a,10b,11,12,12a-dodecahydro-1,6a,8,10a-tetramethyl-4*H*-1,4a-propano-2*H*-phenanthro[1,2-c]pyran-7-carboxylic acid.

SCY-078 (MK-3118) is an investigational antifungal agent currently in clinical development for the treatment of invasive infections caused by Candida and Aspergillus spp. SCY-078 is a novel, first-in-class, semisynthetic derivative of the naturally occurring hemiacetal triterpene glycoside enfumafungin that incorporates a pyridine triazole at position 15 of the core phenanthropyran carboxylic acid ring system and a 2-amino-2,3,3-trimethyl-butyl ether at position 14 (Fig. 1) to enhance its antifungal potency and pharmacokinetic (PK) properties. *In vitro*, SCY-078 has demonstrated potent, broad-spectrum activity against multiple clinical isolates of Candida spp. (2) and isolates of Aspergillus spp. (3), including those with resistance to azoles or ECHs, the latter containing mutations in the *fks* gene(s) (4).

Like the ECHs, SCY-078 disrupts fungal cell wall formation by inhibiting β-1,3-glucan synthesis. The clinical success of this mechanism of action has established ECHs as first-line treatment for invasive candidiasis, although the poor oral bioavailability of ECHs has restricted their clinical utility to parenteral administration. Despite SCY-078 targeting the same fungal site as other ECHs, it is structurally distinct, and in light of its activity against ECH-resistant strains, thought to interact differently at the enzyme target. Thus, SCY-078 distinguishes itself from ECHs by being a first-in-class glucan synthesis inhibitor (GSI) based on enfumafungin, having activity against ECH- and azole-resistant isolates, and being orally bioavailable. It thereby holds promise as an antifungal agent with broad spectrum of activity against Candida and Aspergillus spp. that can be administered both orally and intravenously (i.v.). To date, single- and multiple-dose phase 1 and 2 clinical studies showed that SCY-078 was well tolerated with oral exposure, PK properties, and a safety profile consistent with achieving clinical efficacy (5).

The preclinical PK properties of SCY-078 have been described to support early PK/pharmacodynamic (PD) studies in murine models of disseminated candidiasis (6, 7); however, these studies used a limited number of animals per treatment group and may have only measured exposure after a single oral dose. Here, we present further characterization of SCY-078, including *in vitro* studies to demonstrate properties consistent with oral bioavailability and single- and multiple-dose PK across preclinical species and disposition studies to assess the impact of protein binding on the volume of distribution (*V_d_*_ ss_) for SCY-078 as a predictor of tissue penetration.

Single-dose PK studies were performed in mice, rats, and dogs after oral and i.v. doses to determine PK parameters and bioavailability. In addition, multiple oral-dose studies were conducted in mice to compare the dose-dependent steady-state exposure in plasma and kidney tissue after twice per day (i.e., every 12 h [Q12h]) treatment for 7 days, which reflects dosing regimens used for murine infection models. Lastly, we present preliminary exposure efficacy targets based on free drug the area under the concentration-time curve (AUC)/MIC ratios determined across murine models of disseminated candidiasis.

## RESULTS

### Antifungal activity in a murine model of disseminated candidiasis.

The *in vivo* activity of SCY-078 was evaluated across three murine models of disseminated candidiasis that either evaluated efficacy after 7 days of twice-daily (BID) oral treatment initiated immediately after infection or identified the PK/PD measures associated with a stasis endpoint after either a single dose or the same dose fractionated as half or quarter doses administered 16 h after infection.

### Immediate treatment model.

The *in vivo* efficacy of SCY-078 was evaluated based on a murine model of disseminated candidiasis utilized previously for the characterization of caspofungin (8). SCY-078 demonstrated potent and reproducible activity across four independent studies following BID oral treatment initiated on the day of C. albicans infection with MY1055 (MIC, 0.03 μg/ml). Initial studies demonstrated 100% clearance in kidney fungal burden with SCY-078 doses of ≥12.5 mg/kg. In contrast, treatment with FLU up to 5 mg/kg administered p.o. did not result in clearance of fungal infection in any of the animals tested. Caspofungin was effective in clearing kidney fungal burden at doses of ≥0.125 mg/kg; however, it was administered intraperitoneally because it is not orally bioavailable (data not shown).

Pharmacokinetic analysis was performed in three studies, and the efficacious exposure was defined as that affording complete clearance of measurable infection in >50% of treated animals and a >4-log reduction in CFU in kidney tissue compared to the sham-treated animals (Table 1). The plasma exposure of SCY-078 associated with efficacy expressed as the interstudy mean AUC from 0 to 24 h (AUC_0–24_) ± the standard deviation (SD) was 15.4 ± 2.21 μM·h (11.2 ± 1.61 μg/ml·h).

**TABLE 1:** *In vivo* activity of SCY-078 versus C. albicans MY1055 and target exposures measured after dose 13 on treatment day 7 in a C′5-deficient DBA/2N murine model of disseminated candidiasis

Study and SCY-078 treatment (mg/kg)^*a*^	Plasma AUC_0–24_		Kidney tissue burden (log_10_ CFU/g of tissue)	% animals sterilized^*b*^	Reduction from sham treatment (log_10_ CFU/g of tissue)
	μg·h/ml	μM·h			
Study A					
12.5	19.7	27.0	2.23	80	4.24
Efficacious	12.9	17.7	2.51	60	4
6.25	6.19	8.48	2.78	40	3.69
Study B					
12.5	16.2	22.2	2.2	100	4.67
Efficacious	9.71a	13.3	2.86	50	4.01
6.25	3.27	4.48	3.52	0	3.35
Study C					
6.25	11.0	15.1	2.61	60	4.35
Efficacy (mean)		15.4			

a SCY-078 was administered orally twice daily. Efficacious, projected efficacious exposure assuming linearity regarding both efficacy and plasma exposure. Data are from 3 independent studies.

b Five animals per group.

### Single delayed-dose treatment model.

Single-dose SCY-078 treatment was assessed in a more delayed treatment infection model of disseminated candidiasis where infection with three C. albicans strains (MY1055 at 2.72 × 10^4^ CFU/mouse, CLY724 at 1.71 × 10^4^ CFU/mouse, or CLY18600 at 4.62 × 10^4^ CFU/mouse) was allowed to establish for 16 h (at which point tissue fungal burdens had reached approximately 4 to 4.5 log CFU/g of kidney tissue). Next, a single-dose treatment with SCY-078 was administered. The target endpoint for efficacy in this model was defined as a static effect on the tissue burden measured 96 h after treatment. For comparison, at 96 h the fungal tissue burden had risen in sham treated animals to 6.5 to 7 log_10_ CFU/g of kidney. Regression analysis using SigmaPlot [logarithmic fit *y* = *y*0 + a.ln(*x*)] revealed the stasis endpoints for MY1055, CLY724, and CLY18600 were achieved with SCY-078 doses of 10.28, 9.93, and 8.97 mg/kg, respectively. MY1055 required the highest dose, and linear regression analysis demonstrated an AUC_0–24_ of 14.1 μM·h (10.29 μg/ml·h) achieved the static effect. This exposure is comparable to the therapeutic target (11.2 μg/ml·h; 15.4 μM·h) determined in the immediate treatment model (see above). Caspofungin and fluconazole were not evaluated in this model.

### Fractionated delayed-dose treatment model.

**FIG 2:**
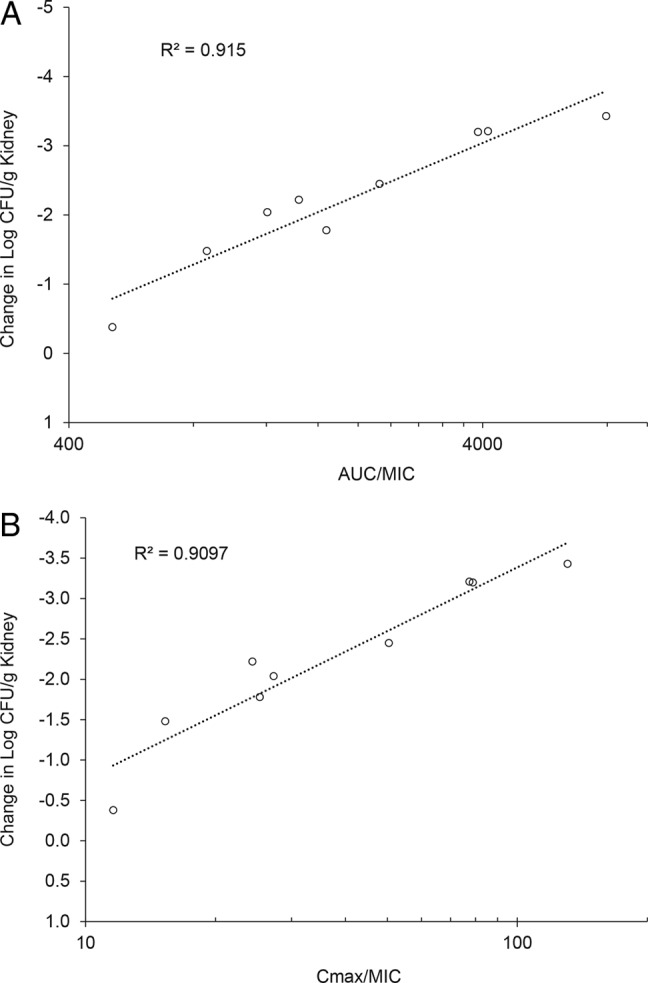
Relationship between SCY-078 PK/PD indices and CFU/g kidney tissue based on the *C*_max_/MIC ratio (A) and the AUC_0–96_/MIC ratio (B). The *C*_max_ values selected were the highest measured concentrations achieved during treatment within each group. The total drug concentration exceeded the MIC [% T MIC] at all PK sampling occasions during the treatment period. PK data were obtained from three animals per time point and dose group on all sampling occasions (0, 0.25, 0.50, 1, 2, 4, 6, 24, 48, 72, and 96 h after the first dose). Changes in the log CFU/g kidney tissue were determined for five animals/group and are expressed relative to animals receiving sham treatment. The lines fitted through the data represent the best fit over the concentrations and doses studied. *R*^2^ is the coefficient of determination. Control (untreated) and sham-treated animals achieved either a mean 4.18 or 5.95 log_10_ increase in CFU/g of kidney tissue during the study.

The fractionated delayed treatment model was used to compare the efficacy of SCY-078 when administered as divided doses of either two half doses or four quarter doses, relative to the outcome of a single dose. The total doses were 12.5, 25, and 50 mg/kg delivered by bolus intraperitoneal injection. Divided doses were administered at either 0 and 48 h or at 0, 24, 48, and 72 h relative to the single dose. Over the range of doses studied the change in CFU/g kidney tissue correlated to *C*_max_/MIC and AUC_0–96_/MIC, with *R*^2^ values of 0.91 and 0.91, respectively (Fig. 2). For all groups, the plasma concentrations were greater than the MIC throughout the treatment period. In all total dose levels the single dose was slightly more efficacious than the divided doses, suggesting that the *C*_max_ is associated with outcome, although the differences did not achieve statistical significance except for the quarter doses of either 6.25 or 3.125 mg/kg relative to the single doses of 25 or 12.5 mg/kg, respectively. Values for the 90% effective dose (ED_90_) were similar for a single dose (5.02 mg/kg) or divided doses (4.40 and 4.25 mg/kg for half or quarter doses); although there were decreases in ED_99_ values with fractionation of dose (i.e., the ED_99_ single dose [13.43 mg/kg] >2 doses [9.83 mg/kg] >4 doses [6.93 mg/kg]). The lowest group mean plasma AUC_0–24_ achieving a static effect relative to pretherapy levels of infection was 13.4 μM·h (9.79 μg/ml·h), which compares favorably with the efficacy targets in both the immediate treatment model (15.4 ± 2.2 μM·h) and the single daily dose-delayed treatment model (14.1 μM·h). Caspofungin and fluconazole were not evaluated in this model.

Overall, SCY-078 demonstrated potent and reproducible activity in each of the murine models of disseminated candidiasis with C. albicans. Therapeutic exposure expressed as plasma AUC_0–24_, was comparable across the tested murine models, i.e., 15.4 ± 2.2, 14.1, and 13.41 μM·h in the immediate-treatment, delayed-single-dose, and delayed-fractionated-dose models, respectively. The mean therapeutic exposure across all models was 14.3 μM·h.

### *In vitro* characterization of SCY-078 for oral administration. (i) *In vitro* permeability and metabolic stability of SCY-078.

Caco-2 cell monolayers were used to assess the *in vitro* absorptive permeability of SCY-078 as a predictor of absorption across the gut following oral delivery. The mean apparent permeability for 5 μM SCY-078 in the apical-to-basolateral direction was 8.9 ± 0.78 ×10^−6^ cm/s (89 ± 7.8 nm/s), indicating good oral absorption since in this assay permeability values of >10^−6^ cm/s are considered predictive of good absorption after oral administration (9).

The metabolic stability of SCY-078 was assessed after incubation with mouse, rat, dog, and human liver microsomes (Table 2) (10). The values for *in vitro* intrinsic clearance (CL_int_) in rodents (mouse and rat), dog, and human liver microsomes were ≤11, ≤48, and 34 μl/min/mg, respectively, indicating that SCY-078 has low clearance with rodent microsomes and low to moderate clearance in dogs and humans. The corresponding values for *in vitro* intrinsic clearance scaled to *in vivo* intrinsic clearance (CL′_int_) in rodent, dog, and human liver microsomes were <40, ≤69, and 38 μl/min/kg, respectively. The half-lives in rodent, dog, and human liver microsomes were ≥125, ≥29, and ≥42 min, respectively. Overall, these data indicate that SCY-078 is most stable in rodent microsomes and most extensively metabolized by dog microsomes. The CL_int_ and half-life values for the control compounds 7-ethoxycoumarin, propranolol, and verapamil were consistent with previous findings in our laboratory and demonstrated the metabolic competency of the microsomes.

**TABLE 2:** Comparison of *in vitro* microsomal metabolism of SCY-078 across species^*a*^

Stability parameter^*b*^	Mouse		Rat (male)	Dog		Human (mixed)
	Male	Female		Male	Female	
% remaining after 30 min	95	88	84	49	84	61
CL_int_ (μl/min/mg)	<10	<10	11	48	15	33
CL′_int_ (μl/min/kg)	<40	<40	20	69	22	38
Half-life (min)	>135	>135	125	29	92	42

a For rats and dogs there were *n* = 2 and for humans there were *n* = 3 independent assays. Pooled microsomes comprised tissue samples from 800, 240, 8, 12, and 50 donors for mice, rats, male dogs, female dogs, and humans, respectively.

b For scaling the *in vitro* CL_int_ to the apparent *in vivo* intrinsic clearance factors, 45 mg of microsomal protein per g of liver was used for rodents (available in the Interspecies Database [http://www.interspeciesinfo.com/]) and humans (10). Assays were performed in triplicate with samples collected after 0, 5, 10, 20, 30, and 45 min of incubation.

### (ii) *In vitro* solubility.

The solubility of SCY-078 was inversely related to pH consistent with pKa values of 9.0 (primary amine), 5.5 (carboxylic acid), and 2.4 (pyridine). At 24 h, amorphous SCY-078 free-base achieved good solubility in simulated gastric fluid (SGF; >5.2 mg/ml) and fed-state intestinal fluid (FeSSIF; >3.0 mg/ml) but was sparingly soluble in fasted-state simulated intestinal fluid (FaSSIF). Solubility improved with the citrate salt form used for i.v. and oral PK studies. SCY-078 salt achieved >20 mg/ml solubility in SGF and FeSSIF at 24 h and >4.2 mg/ml solubility at 24 h in FaSSIF.

### *In vitro* plasma protein binding and blood distribution.

*In vitro* reversible protein binding and blood/plasma partition ratio were determined for SCY-078 in mouse, rat, dog, and human plasma and whole blood (Table 3). The extent of protein binding was high, as anticipated for a lipophilic compound (logD ∼ 6.4 at pH 7.3 and 4.90 at PI). The unbound fraction of SCY-078 in plasma determined by means of equilibrium dialysis was 0.2 to 0.5% (99.8 to 99.5% bound) and appeared to be independent of concentration over the range studied (0.1 to 10 μM). Within the limitations of the assay method, binding perhaps appeared modestly higher in rodent plasma than in dog or human plasma; however, the study was not powered to identify differences between species. The mean blood/plasma partition ratio across species was 0.64 ± 0.079 over the same concentration range, indicating that SCY-078 associates primarily with the plasma compartment of whole blood.

**TABLE 3:** *In vitro* disposition of [^3^H]SCY-078 in DBA mouse, Han Wistar rat, beagle dog, and human blood^*a*^

Species	SCY-078 (μM)	Plasma protein-binding fraction unbound (%)	Blood distribution (blood/plasma ratio)
Mouse	0.1	0.2	0.7
	1	0.2	0.7
	10	0.2	0.6
Rat	0.1	0.2	0.7
	1	0.3	0.7
	10	0.2	0.7
Dog	0.1	0.4	0.5
	1	0.5	0.6
	10	0.3	0.5
Human	0.1	0.2	0.6
	1	0.3	0.7
	10	0.4	0.7

a The total recovery of radioactivity was >95%.

### Pharmacokinetics after intravenous and oral administration to rodents and dogs.

**FIG 3:**
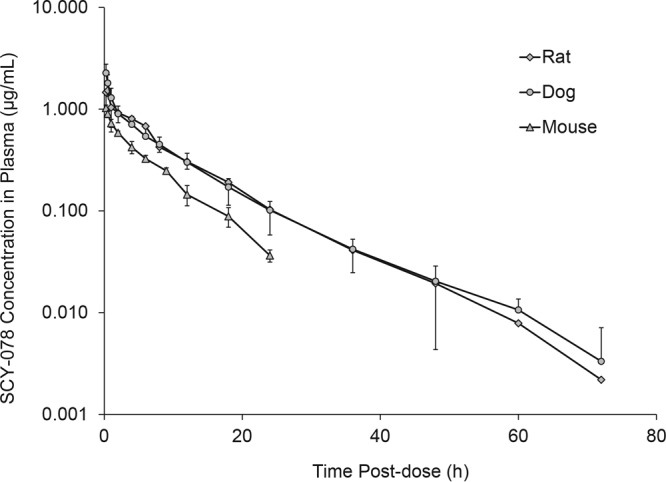
Concentration of SCY-078 in plasma versus time postdose after i.v. administration to treatment-naive female CD-1 mice, male and female Wistar Han rats, and male and female beagle dogs. The data for rats and dogs are plotted as composite mean values for both genders since there was no apparent gender-related difference in pharmacokinetics. Data for mice are normalized to a 5-mg/kg dose.

Summary pharmacokinetic parameters determined in plasma after administration of SCY-078 to rodents and dogs by i.v. and oral routes are presented in Tables 4 and 5, respectively. After i.v. administration to mice (1 mg/kg), rats (5 mg/kg), and dogs (5 mg/kg), SCY-078 demonstrated half-life values of approximately 5.5, 8.7, and 9.3 h, respectively; systemic clearance (CL) values of 0.68, 0.44, and 0.45 liter/h/kg, respectively; and volume of distribution (*V_d_*_ ss_) values of 5.3, 4.7, and 5.1 liters/kg, respectively (Table 4). Plasma concentrations declined in a linear manner in all three species (Fig. 3). A dose normalized AUC_0–∞_ to a 1-mg/kg dose was 1.4 μg/ml·h in mice and ∼2-fold higher in rats and dogs (∼2.4 μg/ml·h).

**TABLE 4:** PK parameters for SCY-078 in plasma after i.v. administration to uninfected, treatment-naive female CD-1 mice, male and female Wistar Han rats, and male and female beagle dogs^*a*^

Species	Dose (mg/kg)	AUC (μg·h/ml)		*t*_1/2_ (h)	CL (liters/h/kg)	*V_d_*_ss_ (liters/kg)	No. of animals	
		AUC_0–24_	AUC_0–∞_				Animals/sex/group/time point	Animals/time point/group
Mouse	1	1.39	1.46	5.49	0.68	5.26	5^*b*^	5
Rat	5	10.3	11.7	8.7	0.44	4.7	4	8
Dog	5	10.4 ± 1.9	11.9 ± 2.2	9.3	0.45	5.1	3	6

a Data for rats and dogs are presented as composite mean values for both genders since there was no apparent gender-related difference in pharmacokinetics.

b Females only.

**TABLE 5:** PK parameters for SCY-078 in plasma after oral administration to uninfected, treatment-naive female CD-1 mice, male and female Wistar Han rats, and male and female beagle dogs^*a*^

Species	Dose (mg/kg)	Days of treatment (dose no.)^*b*^	*C*_max_ (μg/ml)	*T*_max_ (h)	AUC (μg·h/ml)			*t*_1/2_ (h)	Bioavailability (%)^*c*^	No. of animals	
					AUC_0–12_	AUC_0–24_	AUC_0–∞_			Animal/sex/group/time point^*d*^	Animals/time point/group
Mouse	6.25	1	0.39	4	3.08				>41†	5‡	5
	6.25	7 (13)*	0.64	4	5.84	8.36	9.56	7.8		5‡	5
	12	1 (1)	0.90	6	7.58				>51†	5‡	5
	12	7 (13)*	1.61	6	15.1	22.5	28.3	8.8		5‡	5
Rat	20	1 (1)	1.01	8		16.4	20.8	9.1	45	4	8
Dog	20	1 (1)	0.81 ± 0.42	4 ± 2		11.5	16.0	15.2	35	3	6

a Data for rats and dogs are presented as composite mean values for both genders since there was no apparent gender-related difference in pharmacokinetics.

b *, PK after dose 13 following 7 days of Q12h oral treatment.

c †, Calculated from the oral AUC_0–12_ versus the i.v. AUC_0–∞_ rather than the AUC_0–∞_ since mice were dosed BID; consequently, AUC_12–∞_ was not determined after the single dose.

d ‡, Females only.

SCY-078 was well absorbed into plasma after oral administration (Table 5). After single oral doses associated with efficacy in murine models of invasive candidiasis, absolute bioavailability values in mice, rats, and dogs were approximately >51, 45, and 35%, respectively; the corresponding half-life values were approximately 8.3, 9.1, and 15.2 h, respectively. Maximum plasma concentrations were generally reached between 4 and 6 h after dosing across all species, likely reflecting an extended absorption phase from the formulation used in these studies. The bioavailability for mice represents an underestimate because the AUC could only be measured to 12 h rather than to infinity because the animals received a second dose immediately after collection of the 12-h sample. The dose normalized AUC_0–12_ across species was between 0.5 and 0.8 μg/ml·h/mg/kg dose (exposure normalized to a 1-mg/kg dose), although variability was observed among dogs.

Comparison of exposures after a single oral dose and 7 days of twice-per-day (Q12h) oral treatment to mice revealed an ∼2-fold increase in *C*_max_ and AUC. The mean values for *C*_max_ and AUC_0–12_ increased from 0.39 ± 0.13 to 0.64 ± 0.12 μg/ml and from 3.11 to 5.84 μg/ml·h after a single 6.25-mg/kg oral dose and from 0.90 ± 0.25 to 1.61 ± 0.36 μg/ml and 7.58 to 15.1 μg/ml·h after a 12-mg/kg dose. The SCY-078 concentrations in plasma predose on days 4 and 7 after a 6.25-mg/kg dose were 0.26 ± 0.04 and 0.37 ± 0.06 μg/ml, and at 4 h postdose they were 0.74 ± 0.17 and 0.64 ± 0.10 μg/ml, respectively. The equivalent values predose on days 4 and 7 after a 12.5-mg/kg dose were 0.88 ± 0.12 μg/ml and 0.85 ± 0.22 μg/ml predose and 1.59 ± 0.54 and 1.49 ± 0.69 μg/ml at 4 h postdose, respectively.

### Biological distribution to kidney tissue after oral delivery to mice.

**FIG 4:**
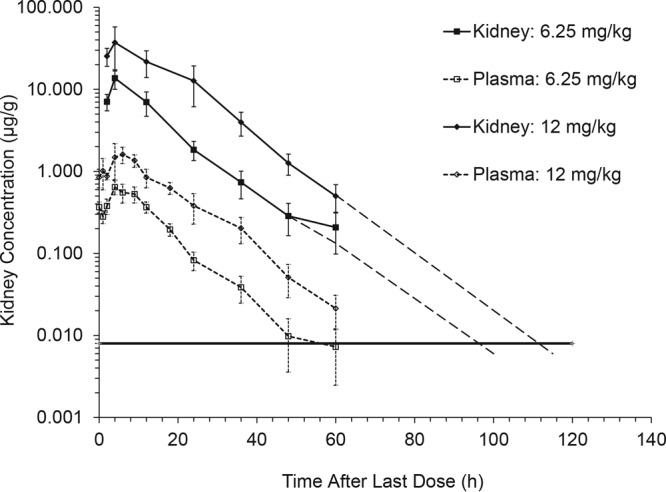
Concentration of SCY-078 in plasma and kidney tissue versus time postdose after 13 Q12h oral doses, reflecting 7 days of treatment to female CD-1 mice (*n* = 5 animals/time point for plasma and kidney). The dashed lines for kidney tissue predict the SCY-078 concentration based on the elimination rate constant calculated between 24 and 60 h after dose 13. The horizontal line represents the lower limit of quantitation determined by LC-MS/MS.

The biological distribution of SCY-078 between plasma and kidney tissue was examined in mice at steady state after oral dose 13 of either 6.25 or 12 mg/kg on day 7 of BID treatment (Table 6). Within each treatment group, kidney exposures, based on the AUC_0–∞_ and *C*_max_ values, were 20- to 25-fold greater than the corresponding parameters for plasma, indicating marked distribution into kidney tissue. The elimination half-lives determined from plasma and kidney tissue were similar (8.3 to 8.9 h, respectively), although the enhanced distribution into kidney tissues would have allowed concentrations to remain measurable to approximately 100 and 115 h for the 6.25- and 12-mg/kg doses, respectively, compared to approximately 50 and 60 h postdose, respectively, in plasma (Fig. 4). The kidney tissue distribution at doses of >12.5 mg/kg BID suggested at least equivalent portioning; however, samples obtained beyond 60 h postdose would be required to accurately determine the AUC in renal tissue.

**TABLE 6:** Comparison of SCY-078 exposures in kidney and plasma in mice measured at steady-state after the oral dose 13 on day 7 of treatment (*n* = 5 animals/time point/treatment group)

Organ or parameter	Oral dose (mg/kg)	*C* (μg/ml)^*a*^		AUC (μg·h/ml)^*b*^			Half-life (h)
		*C*_12_	*C*_max_	AUC_0–12_	AUC_0–24_	AUC_0–∞_	
Kidney	6.25	6.99 ± 2.30	13.7 ± 3.68	118	195	198	9.2
	12	21.7 ± 7.80	37.2 ± 20.5	346	553	700	8.6
Plasma	6.25	0.37 ± 0.06	0.64 ± 0.12	5.84	8.36	9.56	7.8
	12	0.85 ± 0.22	1.61 ± 0.36	15.1	22.52	28.3	8.8
Kidney/plasma ratio	6.25	19.0	21.5	20.0	23.3	20.7	
	12	25.7	23.1	22.9	24.5	24.7	

a The kidney concentrations were measured as μg/g of tissue homogenate; the density of the kidney homogenate was assumed to be 1 g/ml. *C*_12_, *C* at 12 h.

b The PK parameters were calculated from a single composite mean profile for each dose group. The AUC_0–24_ was measured after dose 13 on day 7 of BID oral treatment.

## DISCUSSION

SCY-078 (formerly MK-3118, Fig. 1) is a derivative of enfumafungin and represents the first compound of the triterpene class of β-1,3-glucan synthesis inhibitors to reach clinical development for the treatment of invasive candidiasis. The experiments reported here demonstrate good oral bioavailability of SCY-078 across multiple species and preclinical pharmacokinetic properties consistent with once-per-day oral dosing in humans, together with a high volume of distribution suggestive of excellent tissue distribution. In murine models of disseminated candidiasis, SCY-078 demonstrated consistent anti-Candida activity following oral administration, and the preclinical exposure targets and PK/PD measures associated with achieving efficacy endpoints have been defined.

The inhibition of β-(1,3)-d-glucan synthesis is a proven antifungal target in the treatment of pathogenic fungi, including Aspergillus and Candida spp., and is the mechanism of action for the ECH class of antifungal agents (caspofungin, micafungin, and anidulafungin) now used as first-line treatments for invasive candidiasis (1).

Unlike SCY-078, echinocandins are high-molecular-mass cyclic lipopeptides (∼1,200 kDa) and not orally bioavailable. Thus, they are restricted to parenteral delivery, thereby limiting their use in clinical settings where oral treatment would be desirable or as a potential oral step-down agent after initial i.v. therapy. Although the ECHs continue to be generally very effective *in vitro* against Candida and Aspergillus spp., resistance has been on the rise for Candida infections, particularly in C. glabrata (9). SCY-078 retains *in vitro* activity against many ECH-resistant isolates of Candida that contain mutations in the *fks* gene(s) (4); this suggests that SCY-078 shows promise as an oral and i.v. antifungal agent with broad-spectrum anti-Candida activity.

Enfumafungin was identified as having antifungal activity during a natural product screening campaign (11), and SCY-078 was selected for development following a lead optimization program to improve potency, breadth of activity, and drug-like properties including oral bioavailability (12–14). It is well established that oral bioavailability reflects contributions from multiple processes, including solubility at physiologically relevant pHs in the stomach and intestinal lumen, absorptive permeability, and first-pass metabolism.

SCY-078 exhibited pH-dependent solubility, achieving the highest concentrations in acidic media consistent with simulated gastric fluid (solubility in SGF > 20 mg/ml) and intestinal fluids (solubility in FeSSIF, >20 mg/ml; solubility in FaSSIF, >4.2 mg/ml). *In vitro* SCY-078 is readily permeable through Caco-2 monolayers with an apparent permeability coefficient (P_app_) of approximately 8.9 × 10^−6^ ± 0.78 cm/s (89 ± 7.8 nm/s), where values of >10^−6^ cm/s are consistent with drugs that are well absorbed across the intestinal epithelium (9). Incubations performed with liver microsomes demonstrated that SCY-078 undergoes only moderate turnover with ca. 85% remaining (*t*_0.5_, ≥135 min) after 30 min with rodent microsomes and approximately 50% remaining (*t*_0.5_, ≥29 min) with dog or human microsomes. The corresponding values for intrinsic clearance are ≤10 and <48 μl/min/mg for rodents and larger species, respectively. Scaling intrinsic clearance to hepatic clearance based on liver blood flow indicates low clearance in rodents and moderate clearance in dogs and humans (15). These solubility, permeability, and metabolic stability data are consistent with SCY-078 being well absorbed from the intestine and able to achieve clinically relevant exposures following oral delivery.

As predicted by *in vitro* data, i.v. delivery of SCY-078 showed a low level of clearance in rodents representing approximately 10 to 15% of hepatic blood flow (16). In dogs, clearance was modestly higher; although still low, representing approximately 25% of the hepatic blood flow (16). Estimates for the terminal elimination-phase half-life ranged from 5.5 to 8.7 h in rodents to ∼9.3 h in dogs. These values for low clearance and long half-life in preclinical species are consistent with once daily dosing to humans. Interestingly, values for *V_d_*_ ss_ (4.7 to 5.3 liters/kg) were ∼10-fold body water in all species, an observation suggestive of extensive distribution into compartments other than plasma. This was somewhat unanticipated because the binding of SCY-078 to plasma proteins measured by means of equilibrium dialysis was 99.5 to 99.8%, and extensive, high-affinity binding to plasma proteins is usually associated with low values for *V_d_*_ ss_ since the drug is restrictively retained within the vasculature. Consequently, these data indicate that although SCY-078 is bound extensively to plasma proteins, the binding affinity may be weak, thereby allowing distribution to tissues. *In vitro* blood distribution studies indicated that SCY-078 is largely associated with the plasma compartment of whole blood in all species studied (blood/plasma ratio, 0.5 to 0.7), which is desirable because erythrocytes are not a pharmacologically relevant compartment for invasive candidiasis. Moreover, limited association with erythrocytes suggests that the high values for *V_d_*_ ss_ are predictive of excellent distribution into tissues, which is desirable for treating invasive disease.

SCY-078 was well absorbed, demonstrating good oral bioavailable in mice (>51%), rats (45%), and dogs (35%). Estimates for the terminal elimination-phase half-lives ranged from 7.8 to 9.1 h in rodents to 15 h in dogs and were consistent with values following i.v. administration. At the doses studied, the values for *C*_max_ were ∼1 μg/ml after a single dose and generally observed at 4 to 6 h postdose. In mice, the accumulation factors for *C*_max_ and AUC_0–∞_ after 7 days of twice-daily dosing on a Q12h regimen were ∼2-fold. The group mean SCY-078 concentrations were similar in plasma samples collected predose and 4 h postdose following dose 7 on day 4 relative to dose 13 on day 7, indicating that steady-state exposure had been achieved by day 4 of treatment. Moreover, comparable exposures on days 4 and 7 suggests that SCY-078 does not induce drug-metabolizing enzymes responsible for its metabolism.

Efficacy in murine candidiasis models has been associated with the distribution and persistence of antifungals in the key tissues associated with invasive mycoses (kidney, lung, liver, and spleen) (17–19). Prolonged exposure in kidney beyond when measurable levels of drug have been cleared from plasma has also been attributed to the postantifungal effect, where extended protection is observed against infectious challenge posttreatment (19–21). At steady state, after 7 days of BID oral treatment, the exposure of SCY-078 in kidney tissue exceeded plasma by approximately 20- to 25-fold for AUC_0–∞_ and *C*_max_ (Table 6). In comparison, after i.v. delivery to rodents, the values for the kidney/plasma ratios for the ECHs were approximately 2.9 for caspofungin (22) or 2.8 based on the total radioactivity over 24 h (23), 10.7 for anidulafungin (17), and 3.4 for micafungin (24). Comparison of available values for *V_d_*_ ss_ between SCY-078 and the ECHs indicates a correlation with drug distribution into kidney tissue. For SCY-078 the *V_d_*_ ss_ values ranged from 4.7 to 5.3 liters/kg, while for the ECHs the *V_d_*_ ss_ values ranged from 0.5 to 2.16 liters/kg for anidulafungin, 0.14 to 0.67 liters/kg for caspofungin, and 0.21 to 0.56 liters/kg for micafungin (Table 7). Distribution into erythrocytes likely contributes to the higher *V_d_*_ ss_ value for anidulafungin compared to the other ECHs (17).

**TABLE 7:** Volume of distribution for SCY-078 and echinocandins after i.v. administration

Species	*V_d_*_ss_ (liters/kg)^*a*^			
	SCY-078	Anidulafungin	Caspofungin	Micafungin
Mouse	5.3	NA	0.25 (22), 0.45 (25)	0.38–0.49 (26)
Rat	4.7	2.16^*b*^	0.27 (22), –0.67 (25, 27)	0.42–0.56 (26)
Dog	5.1	NA	NA	0.23–0.25 (26)
Rabbit	NA	0.93–1.23 (28)	0.26 (29), 0.28 (25)	0.313 (30)
Rhesus monkey	4.4	NA	0.16 (22), –0.30 (25)	NA
Human	NA	0.5 (31); 0.57 (32)	0.14 (31)	0.21–0.24 (31); 0.26 (32)

a Source references for various data are indicated within parentheses. Preclinical data were also summarized by Gumbo et al. (33). NA, not applicable.

b Calculated using previously published data (17).

The PK/PD parameters *C*_max_/MIC and AUC/MIC were found to best correlate with outcome following a comparison of single and fractionated doses in the current efficacy studies and in those performed by Andes et al. (34) and Lepak et al. (6) for GSIs in a murine neutropenic model of disseminated candidiasis. This is consistent with general observations for ECH GSIs where, although *C*_max_/MIC does correlate with efficacy, the AUC/MIC is considered a more predictive PK/PD measure, perhaps because it correlates more closely to the more consistent exposure achieved in key tissues at steady state (19, 35).

Four independent studies have been performed in the immediate-treatment model, and SCY-078 met or exceeded efficacy targets in each of the following 7 days of BID treatment with oral doses of either 6.25 or 12.5 mg/kg of SCY-078 in the “fit-for-purpose” formulation. The mean plasma SCY-078 steady-state AUC_0–24_ required for efficacy ± the SD, across the three studies where PK was measured, was 15.4 ± 2.21 μM·h (11.2 ± 1.61 μg/ml·h), and the corresponding total drug AUC/MIC ratio was 373 ± 54. PD studies with the ECH GSIs (34) have indicated that the free-drug AUC/MIC (fAUC/MIC) may be a more reliable predictor of efficacy when comparing therapeutic targets for a given isolate across compounds or when making predictions between species (36–38). The total AUC/MIC ratio of SCY-078 with MY1055 (*in vitro* MIC, 0.03 μg/ml) is equivalent to a free-drug (f) AUC_0–24_ of 0.0224 ± 0.0032 μg/ml·h (f_b_ [fraction bound], 99.8% in mouse plasma) and corresponds to a target fAUC_0–24_/MIC ratio in plasma of ∼0.75 ± 0.107. Recently Lepak et al. (6) evaluated the efficacy of SCY-078 in a murine neutropenic invasive candidiasis model following infections with four C. albicans isolates, four C. glabrata isolates, and three C. parapsilosis isolates, covering an *in vitro* MIC range of 0.03 to 0.25 μg/ml. SCY-078 afforded potent activity *in vivo* against each isolate, and the mean fAUC/MIC ratio for stasis was 0.70 ± 0.54 (median, 0.60; range, 0.10 to 1.71), which is generally consistent with the ratio determined in the present study for MY1055 (0.75 ± 0.107). Interestingly, a C. glabrata isolate (CG 5592) and a C. parapsilosis isolate (CP 20519.069) with the highest MIC values (0.25 μg/ml) required the lowest fAUC/MIC ratios (0.11 and 0.10, respectively) to meet the stasis endpoints, indicating some degree of variability in the fAUC/MIC target between isolates. The total drug AUC_0–24_ target for these two isolates was ∼18.8 μM·h. In a comparison to ECHs that target the same fungal enzyme as SCY-078, the therapeutic efficacy, based on a stasis endpoint, was typically observed when the fAUC/MIC values were 11 to 28 for anidulafungin, 3 to 22 for caspofungin, or 4 to 13 for micafungin (34). When tested by Lepak et al. (6), the range in fAUC/MIC values for SCY-078 against a representative subset of the same panel of isolates (*n* = 11 of 30 isolates) was 0.1 to 1.7 (6). Although further work is required to elucidate the mechanistic basis for the lower fAUC/MIC value with SCY-078, the current data suggest that the fAUC/MIC measured in plasma is lower because of enhanced distribution into tissues, as indicated by the markedly higher *V_d_*_ ss_ and the higher kidney/plasma exposures determined for SCY-078 relative to the echinocandins.

Overall, these studies have identified key attributes that may result in a clinical benefit when SCY-078 is used in the treatment of Candida infection, including low to moderate clearance, oral bioavailability, and tolerability across preclinical species. It demonstrates a high volume of distribution, indicating extensive tissue penetration, as shown by the kidney exposure, and met efficacy endpoints across multiple murine models of invasive candidiasis at concentrations that have been safely achieved after oral administration in humans. Collectively, these data support the further clinical development of SCY-078 for the treatment of infections by Candida spp.

## MATERIALS AND METHODS

### Chemicals.

SCY-078 as a soluble monocitrate salt was prepared by SCYNEXIS (Jersey City, NJ) and was characterized by ^1^H nuclear magnetic resonance and liquid chromatography-mass spectrometry (LC-MS) to demonstrate >99% purity. SCY-078 used for “fit for purpose” formulations were produced by Merck to purity >97%. Control plasma and tissues for bioanalysis were obtained either from satellite (untreated) animals during the murine efficacy studies or purchased as control tissues from Bioreclamation, Inc. (Westbury, NY).

### Murine models of disseminated candidiasis.

The *in vivo* activity of SCY-078 was evaluated using two murine models of disseminated candidiasis to establish the pharmacokinetic exposure target and PK/PD measures associated with efficacy.

In the first model, the target therapeutic exposure was established across four independent experiments, each employing 7 days of twice-daily (BID) oral treatment initiated shortly after infection. To establish the PK/PD parameters associated with the outcome, the efficacy of SCY-078 was compared after single or fractionated doses starting 16 h after the infectious challenge.

A disseminated Candida infection was induced in C′5-deficient DBA/2N mice (Taconic Farms, Germantown, NY), weighing on average 20 g, by i.v. inoculation with C. albicans MY1055 (Merck Culture Collection). C. albicans MY1055 was cultured on Sabouraud dextrose agar (SDA; BBL, Cockeysville, MD) plates at 35°C for 24 h. Yeast cells were washed from the surface of agar plates into sterile saline, and the cell concentrations were quantitated by using a hemocytometer. Viable cell counts were confirmed by a serial 10-fold dilution of the cell suspension and plating on SDA plates. Plates were incubated for 24 to 48 h at 35°C, whereupon the numbers of CFU were determined. The *in vitro* activity of SCY-078 against the MY1055 isolate was evaluated using broth microdilution assays as described in CLSI M27-A3 (39). The MIC endpoints were based on 50% inhibition of fungal growth at 24 h.

For infection, 0.2 ml of a blastospore suspension containing between 2.44 × 10^4^ and 3.56 × 10^4^ CFU of C. albicans MY1055 was inoculated into the lateral tail vein. Mice were housed in groups of up to 10 animals in sterile microisolator cages with sterile bedding. Water and food were provided *ad lib*. The infected and nonmedicated (sham-treated control) animals (*n* = 20) received vehicle only. Treatment groups comprised five animals each, with an additional three animals included for PK analysis for SCY-078. Blood samples were collected from infected satellite PK mice at typically 0, 0.25, 0.5, 1, 2, 4, 6, and 24 h after dose 13 on day 7. Kidneys from five mice were aseptically removed from each treatment group at day 7 after infectious challenge, unless otherwise indicated.

Therapy with SCY-078, caspofungin or fluconazole was initiated within 15 to 30 min after challenge. Mice were treated with SCY-078 with BID p.o. doses of 6.25, 12.5, or 25 mg/kg administered in a “fit-for-purpose” formulation. Caspofungin was administered twice daily via the intraperitoneal route at doses of 0.0078, 0.03, 0.125, and 0.5 mg/kg. Fluconazole was administered p.o. BID at doses of 0.078, 0.31, 1.25, and 5.0 mg/kg (39). At day 7 after challenge, the mice (*n = 5*/group) were euthanized, and both kidneys were aseptically removed, placed in sterile Whirl-Pak bags (Fisher Scientific, Fairlawn, NJ), weighed, and homogenized in 5 ml of sterile physiological saline. Kidney homogenates were serially 10-fold diluted in sterile saline and plated on SDA. Plates were incubated at 35°C and counted after 30 to 48 h of incubation. The CFU/g of kidney were determined, and counts from treatment groups were compared to counts from sham-treated controls using a paired two-tailed *t* test (Microsoft Excel). The percent clearance was determined as the number of mice with no detectable yeast, with a limit of detection of 50 yeast cells per pair of kidneys because of the dilution scheme. For data from individual mice where no detectable yeast were recovered from paired kidneys, 9.8 was entered into a Microsoft Excel spreadsheet formula [log_10_ (5 × raw count)/paired kidney weight)] so that the counts would be one less than the limit of detection or 49 cells per pair of kidneys.

Pharmacokinetic analysis was performed on samples collected from satellite-infected mice (*n = 3*/group) after SCY-078 dose 13 on treatment day 7. Tail bleeds were obtained (20 μl collected into 60 μl of 0.1 M sodium citrate) at time points from 15 min to 6 h, concluding with a terminal bleed at 24 h. Samples were analyzed by liquid chromatography-tandem mass spectrometry (LC-MS/MS) after protein precipitation. Plasma exposure for SCY-078 was calculated from the *in vitro* plasma/whole-blood distribution ratio (see below).

Efficacy was determined in a delayed treatment model based on the protocol described above using typically 5 mice per treatment group. For the single delayed-dose treatment model, a single dose of SCY-078 was administered 16 h after infection. The target endpoint for efficacy in this model was a static effect on the tissue burden measured at 96 h posttreatment. Caspofungin and fluconazole were not evaluated in this model. In the fractionated delayed-dose treatment model, SCY-078 was administered as divided doses of either two half doses or four quarter doses relative to the single dose. The total doses were 12.5, 25, and 50 mg/kg administered as a suspension in a “fit-for-purpose” formulation. Divided doses were administered at either 0 or 48 h or at 0, 24, 48, and 72 h relative to the single dose for the half and quarter doses, respectively. Caspofungin and fluconazole were not evaluated in this model.

### *In vitro* permeability through Caco2 cell monolayers.

Caco2 cells (ATCC CRL-2102) were cultured in Dulbecco modified Eagle medium with the dipeptide form of l-glutamine (GlutaMAX), 10% (vol/vol) fetal bovine serum, and 1% (vol/vol) penicillin-streptomycin at 10,000 U/ml in a 75-ml flask at 37°C in a humidified atmosphere of 5% CO_2_. Near-confluent Caco-2 cell cultures were harvested by trypsinization with 0.25% trypsin at 37°C for 5 min and resuspended in culture medium. The cells were seeded onto semipermeable filter inserts (catalog no. 3401; Corning, Corning, NY) at a density of approximately 200,000 cells/cm^2^. The cell culture medium was changed every 2 to 3 days over a total of 21 days of culture. On the day of the assay, the cell monolayers were rinsed with transport medium (Hanks balanced salt solution with 25 mM glucose and 25 mM HEPES [MediaTech Corning, Tewksbury, MA]), and the absorptive permeation of SCY-078 was evaluated by measuring the flux from the apical to the basolateral compartments. Cell monolayers were incubated with SCY-078 (5 μM) in triplicate for 2 h at 37°C. Samples were removed from the apical and basolateral compartments after incubation and assayed for test compound concentrations by LC-MS/MS. The apparent permeability coefficient (P_app_; cm/s) was calculated as follows: P_app_ = 1/*A*·C_0_ (*dQ*/*dt*), where *dQ*/*dt* is the rate of drug appearance in the basolateral compartment (μmol/s), *C*_0_ is the initial drug concentration in the donor compartment (μM), and *A* is the surface area of the monolayer (cm^2^). The results were expressed as the mean ± the SD from triplicate samples (*n* = 3).

### Solubility.

The solubility of SCY-078 was measured in simulated gastric fluid (SGF), fasted-state simulated intestinal fluid (FaSSIF), and fed-state intestinal fluid (FeSSIF) by Crystal Pharmatech (North Brunswick, NJ). In general, approximately 15 mg of solid was weighed into a 4-ml vial, 3.0 ml of medium was added, and the suspensions were stirred on a rolling incubator (25 rpm) at an ambient room temperature for 24 h. After incubation, 0.5 ml of suspension was centrifuged and filtered (0.45-μm pore size), and the concentration of SCY-078 was determined in the supernatant by high-pressure liquid chromatography (HPLC) with UV detection. The quantity of SCY-078 and the volume of medium was adjusted as required to determine the solubility in each medium.

### *In vitro* metabolic stability in hepatic microsomes.

The metabolic stability of SCY-078 was evaluated with male and female mouse, male rat, male and female dog, and mixed-gender human liver microsomes (XenoTech, Lenexa, KS). SCY-078 (1 μM) was incubated with pooled liver microsomes (0.5 mg protein/ml) for 0, 5, 10, 20, and 30 min at 37°C in the presence of NADPH. Aliquots were taken at each sampling time point and extracted with 5 volumes of ice-cold acetonitrile containing the internal standard (d_9_-SCY-078, 125 ng/ml). Supernatants from the incubation mixtures were analyzed for the parent compound by LC-MS/MS according to the conditions described below. The metabolic competency of microsomal preparations was established using the control compounds 7-ethoxycoumarin, propranolol, and verapamil. The *in vitro* intrinsic clearance (CL_int_), the CL_int_ scaled to the *in vivo* intrinsic clearance (CL′_int_), and the half-life were determined for SCY-078 incubated with microsomes from each species.

### *In vitro* plasma protein binding and blood distribution.

Protein binding of [^3^H]SCY-078 in mouse, rat, dog, and human plasma was determined by means of equilibrium dialysis and liquid scintillation counting. Tritium-labeled SCY-078 in ethanol was added to unlabeled SCY-078 in methanol to prepare stock solutions at 10, 100, and 1,000 μM SCY-078 with constant activity.

Protein binding was determined against isotonic phosphate-buffered saline (pH 7.4) at 37°C for 24 h. Aliquots (10 μl) of each stock solution were added to pooled DBA mouse (*n* = >10 animals), Sprague-Dawley rat, beagle dog, or human plasma (1 ml) to achieve final total concentrations of 0.1, 1, and 10 μM SCY-078. The final concentration of the organic solvent was 0.9% (vol/vol). Dialysis was performed in 1-ml acrylic dialysis cells (Scienceware, South Wayne, NJ), where plasma and buffer were separated by a 12.4-kDa cutoff membrane (Sigma, St. Louis, MO). After incubation, aliquots (100 μl) of plasma and phosphate buffer were counted for radioactivity (Beckman LS6500 liquid scintillation counter), and unbound fractions of SCY-078 were estimated based on the following calculation: unbound fraction = radioactivity (dpm/0.1 ml) in buffer/total radioactivity (dpm/0.1 ml) in plasma.

The blood/plasma partition ratio was determined by adding aliquots of each stock solution to DBA mouse, Sprague-Dawley rat, beagle dog, or human plasma (1 ml) and blood (1 ml) to achieve final concentrations of 0.1, 1, and 10 μM. The final concentration of organic solvent was 0.9% (vol/vol). The resulting blood and plasma samples were incubated at 37°C for 1 h. Blood samples were then subjected to centrifugation at 3,000 rpm for 15 min to obtain plasma. Aliquots (0.1 ml) of plasma samples were counted for radioactivity. The blood/plasma partition ratio was estimated based on comparison of the radioactivity in plasma prepared from the treated blood samples to that in control plasma.

### SCY-078 pharmacokinetics in plasma and kidney tissue.

The pharmacokinetics and bioavailability of SCY-078 were evaluated in uninfected mouse, rat, and dog after p.o. and i.v. administration. The *in vivo* phases of the PK studies were performed at either Avastis (Waltham, MA) or MPI Research (Mattawan, MI) after an Institutional Animal Care and Use Committee (IACUC) review. Avastis and MPI Research are Association for Assessment and Accreditation of Laboratory Animal Care International (AAALAC)-approved facilities. Aqueous formulations of SCY-078 were prepared for p.o. or i.v. delivery, 0.45% (mass/vol) saline or 0.45% (mass/vol) saline containing 2% (mass/vol) PEG400 for i.v. administration and administered at either a 4-, 5-, or 5-ml/kg dose for mice, rats, or dogs, respectively. The formulations used for the PK studies were prepared from an optimized SCY-078 salt form that enhanced the fraction absorbed relative to the fit-for-purpose SCY-078 formulations used for the murine efficacy studies. Blood samples for PK studies were collected into tubes containing K_2_EDTA anticoagulant and stored on wet ice until centrifuged and processed for plasma (stored at approximately −80°C). The peak concentration (*C*_max_), the time to maximum concentration (*T*_max_), the half-life, and the AUC were determined from composite mean plasma concentration-time data for rodents and individual plasma concentration-time data for dogs. All doses and plasma concentrations of SCY-078 are presented as free base.

### Mice.

Female CD-1 mice (*n =* 5/sex/time point/group) weighing approximately 25 g were administered SCY-078 by oral gavage either once daily or as multiple twice-per-day (Q12h) treatments, with individual doses ranging between 3 and 100 mg/kg (6 to 150 mg/kg/day). Mice also received a single 1-mg/ml i.v. dose of SCY-078. Plasma specimens were collected prior to dosing and at 0.25, 0.5, 1, 2, 4, 6, 9, 12, 18, 24, 36, 48, and 60 h after the single i.v. dose and at the same time points from 1 h postdose onward following oral dose 13 on day 7 of treatment. On the first day of oral treatment, plasma specimens were collected immediately before each of the two doses and 1, 2, 4, 6, 9, and 12 h postdosing. Two additional samples (predose and at approximately *T*_max_) were collected after the first dose on day 4 of treatment to determine whether exposure had reached steady-state levels. Kidney tissues were collected at 2, 4, 12, 24, 36, 48, and 60 h after oral dose 13 on treatment day 7, blotted dry, and stored at approximately −80°C until analysis.

### Rats.

Male and female Wistar Han rats (*n =* 3/sex/group/time point) weighing 0.329 to 0.398 kg (males) or 0.187 to 0.244 kg (females) received a single 5-mg/kg dose of SCY-078 as an i.v. injection in the lateral tail administered over 5 min or an oral dose of 20 mg/kg as described above. Blood specimens were collected from four animals/time point/group using a sparse sampling strategy via the sublingual vein at time intervals to 72 h postdosing and processed as described above.

### Dogs.

Treatment naive male and female beagle dogs (*n =* 3/sex/group/time point) weighing 8 to 12 kg (males) or 6 to 10 kg (females) were given a single 5-mg/kg dose of SCY-078 as an i.v. injection over 5 min or an oral dose of 20 mg/kg as described above. Blood specimens were collected from the jugular vein at various time intervals to 72 h postdosing and processed as described above.

### Analysis of SCY-078 in biological samples: mouse kidney tissue homogenization.

On the day of analysis, either treatment-naive (drug-free) kidney tissue or freshly thawed tissue samples from PK studies were weighed and homogenized in 3 volumes of phosphate-buffered saline. The density of kidney tissue used for the determination of volume was assumed to be 1.0 g/ml.

Whole-kidney tissue samples were homogenized in parallel by means of a Precellys 24 bead mill homogenizer (Bertin Technologies, Montigny le Bretonneux, France) using 2-ml Precellys tubes containing 1.4-mm-diameter ceramic beads (CK14) and a single 15-s cycle of 5,500 rpm. Parallel bead homogenization increased throughput and avoided potential cross-contamination of samples since each sample was wholly contained and homogenized within its own sealed vial with no direct contact between the homogenizing instrument or other samples.

### Preparation of biological samples by protein precipitation.

Mouse plasma (50 μl) or kidney tissue homogenate (50 μl) was treated with 5 volumes of acetonitrile containing 50 ng of deuterium (d_9_)-labeled SCY-078 internal standard/ml to precipitate proteins. Treated samples were mixed on a 96-well plate shaker for ∼30 min and then centrifuged at approximately 4,000 × *g* and 15°C for 15 min. Supernatants were transferred to fresh 96-well plates for analysis by LC-MS/MS.

### Preparation of biological samples by liquid-liquid extraction.

Dog and rat plasma (50 μl) were treated with 5.0 μl of d_9_-labeled SCY-078 internal standard (5 μg/ml), followed by 5.0 μl of 1 M KPO_4_ buffer (pH 7). Samples were vortexed briefly before adding 600 μl of methyl-*t*-butyl ether (MtBE). The samples were gently mixed on a 96-well plate shaker for approximately 60 min and then centrifuged at approximately 4,000 × *g* and 4°C for 5 min. The organic (top) layer (500 μl) of each sample was transferred to fresh 96-well plates and evaporated to dryness under N_2_ at 40°C. Residues were reconstituted in 250 μl of acetonitrile-H_2_O (75:25 [vol/vol]) for LC-MS/MS analysis. Calibration standards and quality control samples were prepared in matched drug-free matrix prepared by combining homogenized tissues from treatment-naive animals. Drug-free tissues and plasma were obtained from Bioreclamation.

### HPLC with MS/MS.

All biological samples were assayed by a validated LC-MS/MS method using an HPLC apparatus (Agilent Technologies, Inc., Santa Clara, CA) and an API-4000 triple quadrupole mass spectrometer (Applied Biosystems, Foster City, CA) equipped with a turbo-ion electrospray source operated in positive ionization mode. In summary, treated samples were loaded onto a Phenomenex (Torrance, CA) Kinetix C_18_ analytical column (2.6 μm, 50 by 2.1 mm) and eluted with a gradient mobile phase. Mobile phases consisted of 0.1% (vol/vol) formic acid in water (A) and 0.1% (vol/vol) formic acid in acetonitrile (B). The initial mobile phase composition of 5% B was held for 1.5 min, followed by a linear gradient from 5% to 80% B over 2.5 min. Chromatography was optimized to ensure that PEG400 did not coelute with SCY-078. Test compounds eluted at approximately 3.7 min, and the total run time was 5 min. Instrumental conditions for the mass spectrometer and precursor-to-product ion transitions were selected and optimized for sensitivity and selectivity. The MS/MS transitions for SCY-078 and d_9_-SCY-078 were 730.7/584.5 and 739.7/593.5, respectively. Data acquisition and reduction was performed using Analyst v1.4.2 software (Applied Biosystems).

## References

[1] Pappas PG, Kauffman CA, Andes DR, Clancy CJ, Marr KA, Ostrosky-Zeichner L, Reboli AC, Schuster MG, Vazquez JA, Walsh TJ, Zaoutis TE, Sobel JD 2016 Clinical practice guideline for the management of candidiasis: 2016 update by the Infectious Diseases Society of America. Clin Infect Dis 62:409–417. doi:10.1093/cid/civ1194. 26679628 PMC4725385

[2] Pfaller MA, Messer SA, Motyl MR, Jones RN, Castanheira M 2013 Activity of MK-3118, a new oral glucan synthase inhibitor, tested against *Candida* spp. by two international methods (CLSI and EUCAST). J Antimicrob Chemother 68:858–863. doi:10.1093/jac/dks466. 23190764

[3] Pfaller MA, Messer SA, Motyl MR, Jones RN, Castanheira M 2013 *In vitro* activity of a new oral glucan synthase inhibitor (MK-3118) tested against *Aspergillus* spp. by CLSI and EUCAST broth microdilution methods. Antimicrob Agents Chemother 57:1065–1068. doi:10.1128/AAC.01588-12. 23229479 PMC3553681

[4] Jiménez-Ortigosa C, Paderu P, Motyl MR, Perlin DS 2014 Enfumafungin derivative MK-3118 shows increased *in vitro* potency against clinical echinocandin-resistant *Candida* species and *Aspergillus* species isolates. Antimicrob Agents Chemother 58:1248–1251. doi:10.1128/AAC.02145-13. 24323472 PMC3910825

[5] Trucksis M, Garrett G, Bautmans A, Heirman I, Laethem M, Comisar WA, Maganti L, Bi S, Iwamoto M, O'Mara E, Wagner JA, Depre M, de Hoon J 2011 A phase I multiple-rising dose study evaluating safety, tolerability, and pharmacokinetics of MK-3118, oral glucan synthase inhibitor in healthy volunteers, abstr F1-1390. Abstr 51st Intersci Conf Antimicrob Agents Chemother. American Society for Microbiology, Washington, DC.

[6] Lepak AJ, Marchillo K, Andes DR 2015 Pharmacokinetic target evaluation of a novel oral glucan synthesis inhibitor, SCY-078 (MK3118), using an in vivo murine invasive candidiasis model. Antimicrob Agents Chemother 59:1265–1272. doi:10.1128/AAC.04445-14. 25512406 PMC4335824

[7] Wring S, Abruzzo G, Borroto-Esoda K, Chen Q, Flattery A, Garrett G, Trucksis M, Ribeill Y 2014 Exposure target for the first-in-class, orally active, glucan synthesis inhibitor SCY-078 (MK-3118) in disseminated candidiasis. Abstr 54th Intersci Conf Antimicrob Agents Chemother, abstr F-1595.

[8] Abruzzo GK, Gill CJ, Flattery AM, Kong L, Leighton C, Smith JG, Pikounis VB, Bartizal K, Rosen H 2000 Efficacy of the echinocandin caspofungin against disseminated aspergillosis and candidiasis in cyclophosphamide-induced immunosuppressed mice. Antimicrob Agents Chemother 44:2310–2318. doi:10.1128/AAC.44.9.2310-2318.2000. 10952573 PMC90063

[9] Artursson P, Palm K, Luthman K 2001 Caco-2 monolayers in experimental and theoretical predictions of drug transport. Adv Drug Deliv Rev 46:27–43. doi:10.1016/S0169-409X(00)00128-9. 11259831

[10] Houston JB 1994 Utility of *in vitro* drug metabolism data in predicting *in vivo* metabolic clearance. Biochem Pharmacol 47:1469–1479. doi:10.1016/0006-2952(94)90520-7. 8185657

[11] Peláez F, Cabello A, Platas G, Díez MT, del González VA, Basilio A, Martán I, Vicente F, Bills GE, Giacobbe RA, Schwartz RE, Onish JC, Meinz MS, Abruzzo GK, Flattery AM, Kong L, Kurtz MB 2000 The discovery of enfumafungin, a novel anti-fungal compound produced by an endophytic *Hormonema* species biological activity and taxonomy of the producing organisms. Syst Appl Microbiol 23:333–343. doi:10.1016/S0723-2020(00)80062-4. 11108011

[12] Heasley BH, Pacofsky GJ, Mamai A, Liu H, Nelson K, Coti G, Peel MR, Balkovec JM, Greenlee ML, Liberator P, Meng D, Parker DL, Wilkening RR, Apgar JM, Racine F, Hsu MJ, Giacobbe RA, Kahn JN 2012 Synthesis and biological evaluation of antifungal derivatives of enfumafungin as orally bioavailable inhibitors of β-1,3-glucan synthase. Bioorg Med Chem Lett 22:6811–6816. doi:10.1016/j.bmcl.2012.05.031. 22672801

[13] Peel M, Fan W, Mamai A, Hong J, Orr M, Ouvrey G, Perrey D, Liu H, Jones M, Nelson K, Ogbu C, Lee S, Li K, Kirwan R, Noe A, Sligar A, Martensen P, Balkovec J, Greenlee M, Meng D, Parker D, Wildonger K, Abruzzo G, Flattery A, Galgoci A, Giacobbe R, Gill C, Hsu M, Liberator P, Misura A, Nielsen-Kahn J, Powles MA, Racine F, Dragovic J, Habulihaz B 2010 Enfumafungin derivatives: orally active glucan synthase inhibitors. Abstr 50th Intersci Conf Antimicrob Agents Chemother, abstr F1-845.

[14] Wilkening R, Apgar J, Meng D, Parker D, Greenlee M, Sperbeck D, Wildonger K, Abruzzo G, Flattery A, Galgoci A, Giacobbe R, Gill C, Hsu M, Liberator P, Misura A, Motyl M, Nielsen-Kahn J, Powles MA, Racine F, Dragovic J, Habulihaz B, Fabre E, Fan W, Heasley B, Kirwan R, Lee S, Liu H, Mamai A, Nelson K, Pacofsky G, Peel Balkovec M JM 2010 Enfumafungin derivatives: orally active glucan synthase inhibitors. Abstr 50th Intersci Conf Antimicrob Agents Chemother, abstr F1-846.

[15] Obach RS 1999 Prediction of human clearance of twenty-nine drugs from hepatic microsomal intrinsic clearance data: an examination of in vitro half-life approach and nonspecific binding to microsomes. Drug Metab Dispos 27:1350–1359. 10534321

[16] Davies B, Morris T 1993 Physiological parameters in laboratory animals and humans. Pharm Res 10:1093–1095. doi:10.1023/A:1018943613122. 8378254

[17] Damle B, Stogniew M, Dowell J 2008 Pharmacokinetics and tissue distribution of anidulafungin in rats. Antimicrob Agents Chemother 52:2673–2676. doi:10.1128/AAC.01596-07. 18443124 PMC2443878

[18] Felton T, Troke PF, Hope WW 2014 Tissue penetration of anti-fungal agents. Clin Microbiol Rev 27:68–88. doi:10.1128/CMR.00046-13. 24396137 PMC3910906

[19] Louie A, Deziel M, Liu W, Drusano MF, Gumbo T, Drusano GL 2005 Pharmacodynamics of caspofungin in a murine model of systemic candidiasis: importance of persistence of caspofungin in tissues to understanding drug activity. Antimicrob Agents Chemother 49:5058–5068. doi:10.1128/AAC.49.12.5058-5068.2005. 16304173 PMC1315924

[20] Gumbo T, Drusano GL, Liu W, Ma L, Deziel MR, Drusano MF, Louie A 2006 Anidulafungin pharmacokinetics and microbial response in neutropenic mice with disseminated candidiasis. Antimicrob Agents Chemother 50:3695–3700. doi:10.1128/AAC.00507-06. 16954319 PMC1635198

[21] Hope WW, Drusano GL 2009 Antifungal pharmacokinetics and pharmacodynamics: bridging from the bench to bedside. Clin Microbiol Infect 15:602–612. doi:10.1111/j.1469-0691.2009.02913.x. 19673971

[22] Hajdu R, Thompson R, Sundelof JG, Pelak BA, Bouffard FA, Dropinski JF, Kropp H 1997 Preliminary animal pharmacokinetics of the parenteral antifungal agent MK-0991 (L-743,872). Antimicrob Agents Chemother 41:2339–2344. 9371330 10.1128/aac.41.11.2339 PMC164125

[23] Stone JA, Xu X, Winchell GA, Deutsch PJ, Pearson PG, Migoya EM, Mistry GC, Xi L, Miller A, Sandhu P, Singh R, deLuna F, Dilzer SC, Lasseter KC 2004 Disposition of caspofungin: role of distribution in determining pharmacokinetics in plasma. Antimicrob Agents Chemother 48:815–823. doi:10.1128/AAC.48.3.815-823.2004. 14982770 PMC353127

[24] Niwa T, Yokota Y, Tokunaga A, Yamato Y, Kagayama A, Fujiwara T, Hatakeyama J, Anezaki M, Ohtsuka Y, Takagi A 2004 Tissue distribution after intravenous dosing of micafungin, an antifungal drug, to rats. Biol Pharm Bull 27:1154–1156. doi:10.1248/bpb.27.1154. 15256761

[25] Sandhu P, Xu X, Bondiskey PJ, Balani SK, Morris ML, Tang YS, Miller AR, Pearson PG 2004 Disposition of caspofungin, a novel antifungal agent, in mice, rats, rabbits, and monkeys. Antimicrob Agents Chemother 48:1272–1280. doi:10.1128/AAC.48.4.1272-1280.2004. 15047529 PMC375331

[26] Yamato Y, Kaneko H, Hashimoto T, Katashima M, Ishibashi K, Kawamura A, Terakawa M, Kagayama A 2002 Pharmacokinetics of the anti-fungal drug micafungin in mice, rats and dogs, and its *in vitro* protein binding and distribution to blood cells. Jpn Chemother 50:74–79.

[27] Van Vianen W, de Marie S, ten Kate MT, Mathot RAA, Bakker-Woudenberg IAJM 2006 Caspofungin: antifungal activity *in vitro*, pharmacokinetics, and effects on fungal load and animal survival in neutropenic rats with invasive pulmonary aspergillosis. J Antimicrob Chemother 57:732–740. doi:10.1093/jac/dkl015. 16464895

[28] Groll AH, Mickiene D, Petraitiene R, Petraitis V, Lyman CA, Bacher JS, Piscitelli SC, Walsh TJ 2001 Pharmacokinetic and pharmacodynamic modeling of anidulafungin (LY303366): reappraisal of its efficacy in neutropenic animal models of opportunistic mycoses using optimal plasma sampling. Antimicrob Agents Chemother 45:2845–2855. doi:10.1128/AAC.45.10.2845-2855.2001. 11557479 PMC90741

[29] Groll AH, Gullick BM, Petraitiene R, Petraitis V, Candelario M, Piscitelli SC, Walsh TJ 2001 Compartmental pharmacokinetics of the antifungal echinocandin caspofungin (MK-0991) in rabbits. Antimicrob Agents Chemother 45:596–600. doi:10.1128/AAC.45.2.596-600.2001. 11158761 PMC90333

[30] Groll AH, Mickiene D, Petraitis V, Petraitiene R, Ibrahim KH, Picitelli SC, Bekersky I, Walsh TJ 2001 Compartmental pharmacokinetics and tissue distribution of the antifungal echinocandin lipopeptide micafungin (FK463) in rabbits. Antimicrob Agents Chemother 45:3322–3327. doi:10.1128/AAC.45.12.3322-3327.2001. 11709303 PMC90832

[31] Eschenauer G, DePestel DD, Carver PL 2007 Comparison of echinocandin antifungals. Ther Clin Risk 3:71–97. doi:10.2147/tcrm.2007.3.1.71. PMC1936290 18360617

[32] Denning DW 2003 Echinocandin anti-fungal drugs. Lancet 362:1142–1151. doi:10.1016/S0140-6736(03)14472-8. 14550704

[33] Gumbo T, Ikeda F, Louie A 2007 Glucan synthase inhibitors, p 355–378. *In* Nightingale CH, Ambrose PG, Drusano GL, Murakawa T (ed), Antimicrobial pharmacodynamics in theory and clinical practice, 2nd ed CRC Press, New York, NY.

[34] Andes D, Diekema DJ, Pfaller MA, Bohrmuller J, Marchillo K, Lepak A 2010 *In vivo* comparison of the pharmacodynamic targets for echinocandin drugs against *Candida* species. Antimicrob Agents Chemother 54:2497–2506. doi:10.1128/AAC.01584-09. 20385855 PMC2876357

[35] Andes D 2006 Pharmacokinetics and pharmacodynamics of antifungals. Infect Dis Clin North Am 20:679–697. doi:10.1016/j.idc.2006.06.007. 16984875

[36] Wiederhold NP, Najvar LK, Bocanegra R, Molina D, Olivo M, Graybill JR 2007 *In vivo* efficacy of anidulafungin and caspofungin against *Candida glabrata* and association with in vitro potency in the presence of sera. Antimicrob Agents Chemother 51:1616–1620. doi:10.1128/AAC.00105-07. 17307976 PMC1855556

[37] Ernst EJ, Roling EE, Petzold CR, Keele DJ, Klepser ME 2002 *In vitro* activity of micafungin (FK-463) against *Candida* spp.: microdilution, time-kill, and postantifungal-effect studies. Antimicrob Agents Chemother 46:3846–3853. doi:10.1128/AAC.46.12.3846-3853.2002. 12435687 PMC132786

[38] Paderu P, Garcia-Effron G, Balashov S, Delmas G, Park S, Perlin DS 2007 Serum differentially alters the anti-fungal properties of echinocandin drugs. Antimicrob Agents Chemother 51:2253–2256. doi:10.1128/AAC.01536-06. 17420211 PMC1891414

[39] Clinical and Laboratory Standards Institute. 2008 Reference method for broth dilution antifungal susceptibility testing of yeasts; approved standard M27-A3. Clinical and Laboratory Standards Institute, Wayne, GA.

